# Bis(cyclo­hexyl­ammonium) terephthalate

**DOI:** 10.1107/S1600536812017035

**Published:** 2012-04-28

**Authors:** Meng Ting Han

**Affiliations:** a Ordered Matter Science Research Center, College of Chemistry and Chemical Engineering, Southeast University, Nanjing 211189, People’s Republic of China

## Abstract

In the title mol­ecular salt, 2C_6_H_11_NH_3_^+^·C_8_H_4_O_4_^2−^, the terephthalate dianion is close to being planar (r.m.s. deviation = 0.049 Å). In the crystal, the cations and anions are linked by N—H⋯O hydrogen bonds into (010) sheets. Of the four terephthalate O atoms, two accept two hydrogen bonds each and two accept one hydrogen bond each.

## Related literature

For background to mol­ecular ferroelectric materials, see: Haertling *et al.* (1999 ▶); Homes *et al.* (2001 ▶). For the synthesis of related compounds, see: Fu *et al.* (2009 ▶); Hang *et al.* (2009 ▶).
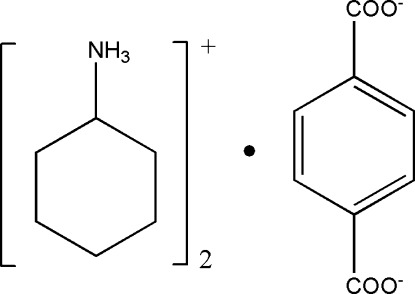


## Experimental

### 

#### Crystal data

2C_6_H_14_N^+^·C_8_H_4_O_4_^2−^
*M* *_r_* = 364.48
Monoclinic, 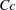

*a* = 11.572 (2) Å
*b* = 22.820 (5) Å
*c* = 8.5426 (17) Å
β = 117.03 (3)°
*V* = 2009.5 (7) Å^3^
*Z* = 4
Mo *K*α radiation
μ = 0.08 mm^−1^
*T* = 293 K
0.33 × 0.28 × 0.20 mm

#### Data collection

Rigaku SCXmini diffractometer
Absorption correction: multi-scan (*CrystalClear*; Rigaku, 2005 ▶) *T*_min_ = 0.973, *T*_max_ = 0.984
8521 measured reflections
2294 independent reflections
2074 reflections with *I* > 2σ(*I*)
*R*_int_ = 0.083

#### Refinement

*R*[*F* ^2^ > 2σ(*F* ^2^)] = 0.084
*wR*(*F* ^2^) = 0.171
*S* = 1.03
2294 reflections
235 parameters
2 restraints
H-atom parameters constrained
Δρ_max_ = 0.22 e Å^−3^
Δρ_min_ = −0.29 e Å^−3^

### 

Data collection: *CrystalClear* (Rigaku, 2005 ▶); cell refinement: *CrystalClear*; data reduction: *CrystalClear*; program(s) used to solve structure: *SHELXS97* (Sheldrick, 2008 ▶); program(s) used to refine structure: *SHELXL97* (Sheldrick, 2008 ▶); molecular graphics: *SHELXTL* (Sheldrick, 2008 ▶); software used to prepare material for publication: *SHELXL97*.

## Supplementary Material

Crystal structure: contains datablock(s) I, global. DOI: 10.1107/S1600536812017035/hb6690sup1.cif

Structure factors: contains datablock(s) I. DOI: 10.1107/S1600536812017035/hb6690Isup2.hkl

Supplementary material file. DOI: 10.1107/S1600536812017035/hb6690Isup3.cml

Additional supplementary materials:  crystallographic information; 3D view; checkCIF report

## Figures and Tables

**Table 1:** Hydrogen-bond geometry (Å, °)

*D*—H⋯*A*	*D*—H	H⋯*A*	*D*⋯*A*	*D*—H⋯*A*
N1—H1*A*⋯O2^i^	0.89	1.88	2.753 (5)	168
N1—H1*B*⋯O3^ii^	0.89	1.90	2.778 (5)	167
N1—H1*D*⋯O4	0.89	1.89	2.766 (5)	168
N2—H2*C*⋯O1^iii^	0.89	1.92	2.786 (5)	162
N2—H2*D*⋯O1^iv^	0.89	2.01	2.827 (5)	152
N2—H2*E*⋯O3^v^	0.89	1.97	2.785 (5)	151

Symmetry codes: (i) 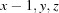; (ii) 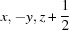; (iii) 
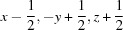
; (iv) 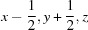; (v) 
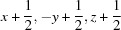
.

**Fig. 1.**
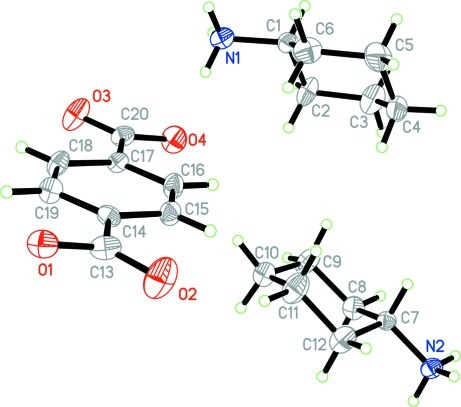
Perspective structure of the title compound, showing displacement ellipsoids drawn at the 30% probability level.

**Fig. 2.**
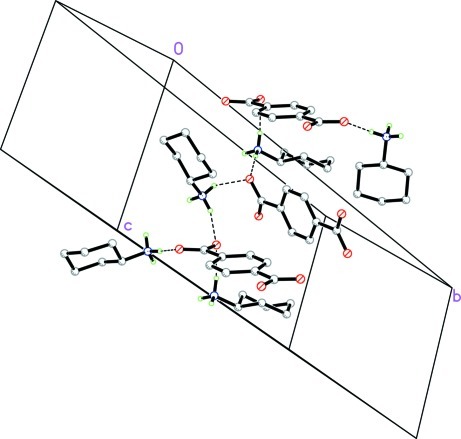
The crystal packing of the title compound viewed along the b axis showing the hydrogen bondings network. Some of the H atoms have been ommitted for clarity.

## supplementary crystallographic information

### Comment

At present, much attention in ferroelectric material field is focused on developing ferroelectric pure organic or inorganic compounds (Haertling *et al.* 1999; Homes *et al.* 2001). Recently we have reported the synthesis of a variety of compounds(Fu *et al.*, 2009; Hang *et al.*, 2009), which have potential piezoelectric and ferroelectric properties. In order to find more dielectric ferroelectric materials, we investigate the physical properties of the title compound(Fig. 1). The dielectric constant of the title compound as a function of temperature indicates that the permittivity is basically temperature-independent (dielectric constant equaling to 0.26 to 0.79), suggesting that this compound should be not a real ferroelectrics or there may be no distinct phase transition occurred within the measured temperature range. Similarly, below the melting point (higher than 533 K) of the compound, the dielectric constant as a function of temperature also goes smoothly, and there is no dielectric anomalyobserved (dielectric constant equaling to 0.26 to 0.79). Herein, we report the synthesis and crystal structure of the title compound.

The molecular structure of the title compound is shown in Fig. 1. There are one C_8_H_4_O_4_^2-^ anion and two cyclohexanamine cation in the asymmetric unit. All cyclohexanamine rings are, of course, chair conformation. As can be seen from the packing diagram (Fig. 2), molecules are connected *via* intermolecular N—H···O hydrogen bonds to form a twodimensional plane. Dipole–dipole and van der Waals interactions are effective in the molecular packing.

### Experimental

A mix of cyclohexylamine (0.461 g, 0.01 mol) and terephthalic acid (0.8307 g, 0.005 mol) in water (20 ml) was stirred until clear. After several days, the title compound was formed and recrystallized from solution to afford colourless prisms.

### Refinement

H atoms were positioned geometrically and refined using a riding model, with C—H(aromatic) = 0.93 and 0.97 (methylene) Å, N—H = 0.89 Å and with *U*_iso_(H) = 1.2*U*_eq_(C) and *U*_iso_(H) = 1.5*U*_eq_(N).

### Crystal data

**Table:**

2C_6_H_14_N^+^·C_8_H_4_O_4_^2^^−^	*F*(000) = 792
*M**_r_* = 364.48	*D*_x_ = 1.205 Mg m^−^^3^
Monoclinic, *C**c*	Mo *K*α radiation, λ = 0.71073 Å
Hall symbol: C -2yc	Cell parameters from 4397 reflections
*a* = 11.572 (2) Å	θ = 2.3–27.5°
*b* = 22.820 (5) Å	µ = 0.08 mm^−^^1^
*c* = 8.5426 (17) Å	*T* = 293 K
β = 117.03 (3)°	Prism, colourless
*V* = 2009.5 (7) Å^3^	0.33 × 0.28 × 0.20 mm
*Z* = 4	

### Data collection

**Table:**

Rigaku SCXmini diffractometer	2074 reflections with *I* > 2σ(*I*)
Radiation source: fine-focus sealed tube	*R*_int_ = 0.083
Graphite monochromator	θ_max_ = 27.5°, θ_min_ = 3.2°
ω scans	*h* = −15→15
Absorption correction: multi-scan (*CrystalClear*; Rigaku, 2005)	*k* = −29→29
*T*_min_ = 0.973, *T*_max_ = 0.984	*l* = −11→11
8521 measured reflections	2 standard reflections every 150 reflections
2294 independent reflections	intensity decay: none

### Refinement

**Table:**

Refinement on *F*^2^	Primary atom site location: structure-invariant direct methods
Least-squares matrix: full	Secondary atom site location: difference Fourier map
*R*[*F*^2^ > 2σ(*F*^2^)] = 0.084	Hydrogen site location: inferred from neighbouring sites
*wR*(*F*^2^) = 0.171	H-atom parameters constrained
*S* = 1.03	*w* = 1/[σ^2^(*F*_o_^2^) + (0.0494*P*)^2^ + 0.9408*P*] where *P* = (*F*_o_^2^ + 2*F*_c_^2^)/3
2294 reflections	(Δ/σ)_max_ < 0.001
235 parameters	Δρ_max_ = 0.22 e Å^−^^3^
2 restraints	Δρ_min_ = −0.29 e Å^−^^3^

### Special details

**Table:**

Geometry. All e.s.d.'s (except the e.s.d. in the dihedral angle between two l.s. planes) are estimated using the full covariance matrix. The cell e.s.d.'s are taken into account individually in the estimation of e.s.d.'s in distances, angles and torsion angles; correlations between e.s.d.'s in cell parameters are only used when they are defined by crystal symmetry. An approximate (isotropic) treatment of cell e.s.d.'s is used for estimating e.s.d.'s involving l.s. planes.
Refinement. Refinement of *F*^2^ against ALL reflections. The weighted *R*-factor *wR* and goodness of fit *S* are based on *F*^2^, conventional *R*-factors *R* are based on *F*, with *F* set to zero for negative *F*^2^. The threshold expression of *F*^2^ > σ(*F*^2^) is used only for calculating *R*-factors(gt) *etc*. and is not relevant to the choice of reflections for refinement. *R*-factors based on *F*^2^ are statistically about twice as large as those based on *F*, and *R*- factors based on ALL data will be even larger.

### Fractional atomic coordinates and isotropic or equivalent isotropic displacement parameters (Å^2^)

**Table:**

	*x*	*y*	*z*	*U*_iso_*/*U*_eq_	
O1	1.0562 (3)	0.01965 (14)	0.2071 (4)	0.0459 (9)	
O2	1.1042 (3)	0.09972 (18)	0.3698 (5)	0.0703 (13)	
O3	0.4191 (3)	0.03397 (15)	0.1026 (5)	0.0587 (11)	
O4	0.4746 (3)	0.11090 (15)	0.2780 (5)	0.0534 (10)	
C13	1.0266 (5)	0.0614 (2)	0.2818 (7)	0.0415 (13)	
C14	0.8887 (4)	0.0637 (2)	0.2555 (6)	0.0310 (11)	
C15	0.8548 (4)	0.1042 (2)	0.3456 (6)	0.0369 (12)	
H15A	0.9168	0.1306	0.4196	0.044*	
C16	0.7307 (5)	0.1067 (2)	0.3288 (6)	0.0395 (13)	
H16A	0.7101	0.1342	0.3924	0.047*	
C17	0.6362 (4)	0.06814 (19)	0.2172 (6)	0.0292 (11)	
C18	0.6711 (4)	0.0271 (2)	0.1269 (6)	0.0392 (13)	
H18A	0.6094	0.0005	0.0533	0.047*	
C19	0.7956 (4)	0.0247 (2)	0.1436 (6)	0.0380 (12)	
H19A	0.8168	−0.0028	0.0804	0.046*	
C20	0.4989 (5)	0.0710 (2)	0.1974 (7)	0.0377 (13)	
N1	0.3672 (3)	0.08243 (16)	0.4988 (5)	0.0406 (10)	
H1A	0.2811	0.0828	0.4535	0.061*	
H1B	0.3965	0.0464	0.5355	0.061*	
H1D	0.3903	0.0934	0.4169	0.061*	
C1	0.4234 (4)	0.1238 (2)	0.6496 (6)	0.0383 (12)	
H1C	0.3964	0.1115	0.7379	0.046*	
C2	0.3719 (5)	0.1835 (2)	0.5880 (8)	0.0530 (15)	
H2A	0.2782	0.1831	0.5413	0.064*	
H2B	0.3922	0.1949	0.4939	0.064*	
C3	0.4286 (5)	0.2279 (3)	0.7349 (8)	0.0670 (18)	
H3A	0.3993	0.2194	0.8223	0.080*	
H3B	0.3981	0.2668	0.6884	0.080*	
C4	0.5761 (5)	0.2269 (3)	0.8212 (7)	0.0594 (16)	
H4A	0.6058	0.2401	0.7377	0.071*	
H4B	0.6100	0.2535	0.9204	0.071*	
C5	0.6263 (6)	0.1663 (3)	0.8828 (7)	0.0693 (18)	
H5A	0.6036	0.1546	0.9745	0.083*	
H5B	0.7201	0.1662	0.9321	0.083*	
C6	0.5696 (5)	0.1222 (2)	0.7315 (7)	0.0562 (15)	
H6A	0.5980	0.1318	0.6438	0.067*	
H6B	0.6003	0.0831	0.7753	0.067*	
N2	0.6584 (3)	0.43904 (15)	0.4889 (5)	0.0374 (10)	
H2C	0.6172	0.4577	0.5404	0.056*	
H2D	0.6397	0.4560	0.3863	0.056*	
H2E	0.7436	0.4408	0.5578	0.056*	
C7	0.6162 (4)	0.37641 (19)	0.4591 (6)	0.0324 (11)	
H7A	0.6378	0.3584	0.5732	0.039*	
C8	0.4710 (4)	0.3733 (2)	0.3475 (7)	0.0457 (13)	
H8A	0.4476	0.3929	0.2363	0.055*	
H8B	0.4283	0.3936	0.4067	0.055*	
C9	0.4243 (5)	0.3097 (2)	0.3133 (8)	0.0601 (17)	
H9A	0.4364	0.2920	0.4229	0.072*	
H9B	0.3322	0.3091	0.2331	0.072*	
C10	0.4958 (6)	0.2740 (2)	0.2362 (7)	0.0650 (18)	
H10A	0.4720	0.2876	0.1179	0.078*	
H10B	0.4700	0.2333	0.2292	0.078*	
C11	0.6422 (5)	0.2788 (2)	0.3465 (9)	0.0646 (17)	
H11A	0.6851	0.2581	0.2883	0.078*	
H11B	0.6674	0.2604	0.4598	0.078*	
C12	0.6860 (5)	0.3432 (2)	0.3739 (8)	0.0565 (15)	
H12A	0.7790	0.3452	0.4481	0.068*	
H12B	0.6670	0.3609	0.2615	0.068*	

### Atomic displacement parameters (Å^2^)

**Table:**

	*U*^11^	*U*^22^	*U*^33^	*U*^12^	*U*^13^	*U*^23^
O1	0.042 (2)	0.047 (2)	0.055 (2)	0.0090 (18)	0.0274 (19)	0.0070 (19)
O2	0.034 (2)	0.070 (3)	0.101 (4)	−0.014 (2)	0.026 (2)	−0.027 (3)
O3	0.031 (2)	0.043 (2)	0.104 (3)	−0.0052 (18)	0.031 (2)	−0.021 (2)
O4	0.049 (2)	0.056 (2)	0.073 (3)	0.0003 (18)	0.043 (2)	−0.011 (2)
C13	0.032 (3)	0.042 (3)	0.052 (4)	0.004 (3)	0.019 (3)	0.012 (3)
C14	0.027 (3)	0.039 (3)	0.030 (3)	0.004 (2)	0.015 (2)	0.005 (2)
C15	0.033 (3)	0.040 (3)	0.036 (3)	−0.008 (2)	0.014 (2)	−0.008 (2)
C16	0.039 (3)	0.040 (3)	0.045 (3)	−0.004 (2)	0.024 (3)	−0.012 (3)
C17	0.024 (2)	0.028 (3)	0.039 (3)	0.001 (2)	0.018 (2)	−0.001 (2)
C18	0.031 (3)	0.033 (3)	0.049 (3)	−0.007 (2)	0.013 (3)	−0.008 (2)
C19	0.028 (3)	0.041 (3)	0.044 (3)	0.001 (2)	0.015 (2)	−0.004 (2)
C20	0.033 (3)	0.036 (3)	0.049 (4)	0.000 (3)	0.023 (3)	0.003 (3)
N1	0.033 (2)	0.039 (2)	0.054 (3)	0.0005 (19)	0.023 (2)	0.002 (2)
C1	0.035 (3)	0.039 (3)	0.039 (3)	−0.006 (2)	0.016 (2)	−0.001 (2)
C2	0.037 (3)	0.038 (3)	0.068 (4)	−0.001 (2)	0.009 (3)	−0.016 (3)
C3	0.058 (4)	0.057 (4)	0.082 (5)	−0.004 (3)	0.028 (4)	−0.015 (4)
C4	0.070 (4)	0.061 (4)	0.050 (4)	−0.025 (3)	0.030 (3)	−0.017 (3)
C5	0.050 (4)	0.087 (5)	0.046 (4)	−0.009 (3)	0.001 (3)	0.006 (4)
C6	0.038 (3)	0.060 (4)	0.057 (4)	0.006 (3)	0.010 (3)	0.012 (3)
N2	0.032 (2)	0.037 (2)	0.045 (3)	−0.0012 (19)	0.020 (2)	0.000 (2)
C7	0.033 (3)	0.033 (3)	0.034 (3)	0.001 (2)	0.016 (2)	0.001 (2)
C8	0.038 (3)	0.052 (3)	0.050 (3)	0.000 (3)	0.022 (3)	−0.005 (3)
C9	0.051 (3)	0.046 (4)	0.077 (5)	−0.014 (3)	0.023 (3)	−0.009 (3)
C10	0.090 (5)	0.050 (4)	0.055 (4)	−0.017 (4)	0.032 (4)	−0.009 (3)
C11	0.059 (4)	0.045 (4)	0.090 (5)	0.002 (3)	0.034 (4)	−0.013 (3)
C12	0.051 (3)	0.048 (3)	0.079 (4)	−0.002 (3)	0.036 (3)	−0.008 (3)

### Geometric parameters (Å, º)

**Table:**

O1—C13	1.278 (6)	C4—C5	1.500 (8)
O2—C13	1.234 (6)	C4—H4A	0.9700
O3—C20	1.242 (6)	C4—H4B	0.9700
O4—C20	1.249 (6)	C5—C6	1.530 (7)
C13—C14	1.508 (6)	C5—H5A	0.9700
C14—C15	1.369 (6)	C5—H5B	0.9700
C14—C19	1.389 (6)	C6—H6A	0.9700
C15—C16	1.378 (6)	C6—H6B	0.9700
C15—H15A	0.9300	N2—C7	1.495 (5)
C16—C17	1.389 (6)	N2—H2C	0.8900
C16—H16A	0.9300	N2—H2D	0.8900
C17—C18	1.385 (6)	N2—H2E	0.8900
C17—C20	1.521 (6)	C7—C8	1.510 (6)
C18—C19	1.383 (6)	C7—C12	1.515 (6)
C18—H18A	0.9300	C7—H7A	0.9800
C19—H19A	0.9300	C8—C9	1.531 (6)
N1—C1	1.487 (5)	C8—H8A	0.9700
N1—H1A	0.8900	C8—H8B	0.9700
N1—H1B	0.8900	C9—C10	1.509 (7)
N1—H1D	0.8900	C9—H9A	0.9700
C1—C2	1.485 (6)	C9—H9B	0.9700
C1—C6	1.509 (7)	C10—C11	1.523 (7)
C1—H1C	0.9800	C10—H10A	0.9700
C2—C3	1.511 (7)	C10—H10B	0.9700
C2—H2A	0.9700	C11—C12	1.537 (7)
C2—H2B	0.9700	C11—H11A	0.9700
C3—C4	1.520 (7)	C11—H11B	0.9700
C3—H3A	0.9700	C12—H12A	0.9700
C3—H3B	0.9700	C12—H12B	0.9700
			
O2—C13—O1	123.1 (5)	C4—C5—C6	111.2 (4)
O2—C13—C14	119.6 (5)	C4—C5—H5A	109.4
O1—C13—C14	117.2 (5)	C6—C5—H5A	109.4
C15—C14—C19	119.2 (4)	C4—C5—H5B	109.4
C15—C14—C13	119.6 (4)	C6—C5—H5B	109.4
C19—C14—C13	121.2 (4)	H5A—C5—H5B	108.0
C14—C15—C16	121.4 (5)	C1—C6—C5	109.6 (5)
C14—C15—H15A	119.3	C1—C6—H6A	109.7
C16—C15—H15A	119.3	C5—C6—H6A	109.7
C15—C16—C17	120.2 (4)	C1—C6—H6B	109.7
C15—C16—H16A	119.9	C5—C6—H6B	109.7
C17—C16—H16A	119.9	H6A—C6—H6B	108.2
C18—C17—C16	118.2 (4)	C7—N2—H2C	109.5
C18—C17—C20	121.4 (4)	C7—N2—H2D	109.5
C16—C17—C20	120.4 (4)	H2C—N2—H2D	109.5
C19—C18—C17	121.5 (4)	C7—N2—H2E	109.5
C19—C18—H18A	119.2	H2C—N2—H2E	109.5
C17—C18—H18A	119.2	H2D—N2—H2E	109.5
C18—C19—C14	119.5 (4)	N2—C7—C8	109.6 (3)
C18—C19—H19A	120.3	N2—C7—C12	110.9 (4)
C14—C19—H19A	120.3	C8—C7—C12	110.9 (4)
O3—C20—O4	124.7 (5)	N2—C7—H7A	108.5
O3—C20—C17	118.2 (4)	C8—C7—H7A	108.5
O4—C20—C17	117.1 (4)	C12—C7—H7A	108.5
C1—N1—H1A	109.5	C7—C8—C9	111.1 (4)
C1—N1—H1B	109.5	C7—C8—H8A	109.4
H1A—N1—H1B	109.5	C9—C8—H8A	109.4
C1—N1—H1D	109.5	C7—C8—H8B	109.4
H1A—N1—H1D	109.5	C9—C8—H8B	109.4
H1B—N1—H1D	109.5	H8A—C8—H8B	108.0
C2—C1—N1	109.2 (4)	C10—C9—C8	112.3 (5)
C2—C1—C6	111.8 (4)	C10—C9—H9A	109.1
N1—C1—C6	110.1 (4)	C8—C9—H9A	109.1
C2—C1—H1C	108.6	C10—C9—H9B	109.1
N1—C1—H1C	108.6	C8—C9—H9B	109.1
C6—C1—H1C	108.6	H9A—C9—H9B	107.9
C1—C2—C3	111.6 (5)	C9—C10—C11	111.6 (5)
C1—C2—H2A	109.3	C9—C10—H10A	109.3
C3—C2—H2A	109.3	C11—C10—H10A	109.3
C1—C2—H2B	109.3	C9—C10—H10B	109.3
C3—C2—H2B	109.3	C11—C10—H10B	109.3
H2A—C2—H2B	108.0	H10A—C10—H10B	108.0
C2—C3—C4	111.1 (5)	C10—C11—C12	111.1 (5)
C2—C3—H3A	109.4	C10—C11—H11A	109.4
C4—C3—H3A	109.4	C12—C11—H11A	109.4
C2—C3—H3B	109.4	C10—C11—H11B	109.4
C4—C3—H3B	109.4	C12—C11—H11B	109.4
H3A—C3—H3B	108.0	H11A—C11—H11B	108.0
C5—C4—C3	110.8 (5)	C7—C12—C11	109.9 (4)
C5—C4—H4A	109.5	C7—C12—H12A	109.7
C3—C4—H4A	109.5	C11—C12—H12A	109.7
C5—C4—H4B	109.5	C7—C12—H12B	109.7
C3—C4—H4B	109.5	C11—C12—H12B	109.7
H4A—C4—H4B	108.1	H12A—C12—H12B	108.2
			
O2—C13—C14—C15	−7.4 (7)	C16—C17—C20—O4	3.6 (7)
O1—C13—C14—C15	174.0 (4)	N1—C1—C2—C3	−178.4 (4)
O2—C13—C14—C19	173.7 (5)	C6—C1—C2—C3	−56.4 (6)
O1—C13—C14—C19	−4.9 (7)	C1—C2—C3—C4	54.8 (7)
C19—C14—C15—C16	0.7 (7)	C2—C3—C4—C5	−54.9 (7)
C13—C14—C15—C16	−178.2 (4)	C3—C4—C5—C6	56.3 (7)
C14—C15—C16—C17	−0.8 (7)	C2—C1—C6—C5	56.7 (6)
C15—C16—C17—C18	1.1 (7)	N1—C1—C6—C5	178.2 (4)
C15—C16—C17—C20	−179.6 (5)	C4—C5—C6—C1	−56.9 (6)
C16—C17—C18—C19	−1.2 (7)	N2—C7—C8—C9	179.7 (4)
C20—C17—C18—C19	179.5 (4)	C12—C7—C8—C9	56.9 (6)
C17—C18—C19—C14	1.1 (7)	C7—C8—C9—C10	−53.9 (6)
C15—C14—C19—C18	−0.8 (7)	C8—C9—C10—C11	52.6 (7)
C13—C14—C19—C18	178.1 (4)	C9—C10—C11—C12	−54.3 (7)
C18—C17—C20—O3	2.5 (7)	N2—C7—C12—C11	179.3 (4)
C16—C17—C20—O3	−176.7 (5)	C8—C7—C12—C11	−58.6 (6)
C18—C17—C20—O4	−177.1 (5)	C10—C11—C12—C7	57.2 (6)

### Hydrogen-bond geometry (Å, º)

**Table:**

*D*—H···*A*	*D*—H	H···*A*	*D*···*A*	*D*—H···*A*
N1—H1*A*···O2^i^	0.89	1.88	2.753 (5)	168
N1—H1*B*···O3^ii^	0.89	1.90	2.778 (5)	167
N1—H1*D*···O4	0.89	1.89	2.766 (5)	168
N2—H2*C*···O1^iii^	0.89	1.92	2.786 (5)	162
N2—H2*D*···O1^iv^	0.89	2.01	2.827 (5)	152
N2—H2*E*···O3^v^	0.89	1.97	2.785 (5)	151

Symmetry codes: (i) *x*−1, *y*, *z*; (ii) *x*, −*y*, *z*+1/2; (iii) *x*−1/2, −*y*+1/2, *z*+1/2; (iv) *x*−1/2, *y*+1/2, *z*; (v) *x*+1/2, −*y*+1/2, *z*+1/2.
